# Molecular characterisation of foot-and-mouth disease viruses collected from Bangladesh during 2021–23: evidence for trans-pool spread of exotic viral lineages

**DOI:** 10.1186/s13567-025-01655-0

**Published:** 2025-11-27

**Authors:** Hyeonjeong Kang, Soyoon Ryoo, Da-Rae Lim, Tae-Yoon Eom, Jae-Myung Kim, Jongwan Kim, Shukes Chandra Badhy, Mohammad Sadekuzzaman, Shamima Akter, Antonello Di Nardo, Donald P. King, Md. Golam Azam Chowdhury, Sang-Ho Cha

**Affiliations:** 1 Foot-and-Mouth-Disease Research Division, Animal and Plant Quarantine Agency, Gimcheon-si, Gyeongsangbuk-do 39660 Republic of Korea; 2 Animal Virology Laboratory, School of Life Sciences, Kyungpook National University, Daegu, 41566 Republic of Korea; 3 Central Disease Investigation Laboratory (CDIL), 48 Kazi Alauddin Road, Dhaka, 1000 Bangladesh; 4 The Pirbright Institute, Surrey, GU24 0NF UK

**Keywords:** Foot-and-mouth disease virus, serotype O, serotype A, Bangladesh, A/ASIA/Iran-05, trans-pool movement

## Abstract

Foot-and-mouth disease (FMD) remains endemic in Bangladesh with the persistent circulation of FMD virus (FMDV) serotypes O, A, and Asia 1, underscoring the need for robust epidemiological data to inform and optimize national FMD control strategies. This study analyzed 57 VP1 coding sequences obtained from 89 clinical samples collected from FMD-infected cattle in Bangladesh between 2021 and 2023. Phylogenetic analysis classified these field isolates into three FMDV lineages: O/ME-SA/Ind-2001e (*n* = 40, 70.2%), O/ME-SA/SA-2018 (*n* = 15, 26.3%), and A/ASIA/Iran-05 (*n* = 2, 3.5%). The O/ME-SA/SA-2018 lineage detected in 2022 (61.1%) and 2023 (25.0%) shared 94.24–99.06% nucleotide sequence identity with viruses from the same lineage collected in 2021. Sequences for O/ME-SA/SA-2018 were monophyletic, while data for O/ME-SA/Ind-2001e provided evidence for viruses evolving within two sister clades in Bangladesh during 2021–23. Additionally, two samples collected in 2023 and tested positive for serotype A were characterized as belonging to the A/ASIA/Iran-05 lineage (sublineage FAR-11), representing the first cases of this lineage reported within the FMD endemic Pool 2. Analyses showed that VP1 sequences for two isolates (A/BAN5/2023 and A/BAN6/2023) were most closely related to a virus isolated in Pakistan during 2022 (PAK/41/2022a), sharing 97.81% nucleotide identity and a common ancestor dated March 2022. Further studies are needed to identify likely pathways of introduction of the A/Iran-05 lineage in Bangladesh, as well as to assess the potential risk to neighboring countries. This study highlights the importance of continuous FMD monitoring in Bangladesh to inform both control and vaccination strategies.

## Introduction

Foot-and-mouth disease (FMD) is a highly contagious viral infection that affects cloven-hoofed animals, causing severe economic losses to the livestock industry. Vesicular and erosive lesions on the mouth, nose, gums, feet, and teats characterize the disease. It leads to increased mortality in young animals, reduced milk production, stunted growth, and impaired reproduction in adults. The causative agent, FMD virus (FMDV), is a positive-sense single-stranded RNA virus belonging to the genus *Aphthovirus* within the family *Picornaviridae*. FMDV is immunologically classified into seven distinct serotypes [O, A, Asia1, C, Southern African Territories (SAT) 1, SAT 2, and SAT 3], among which there is no cross-protection. The VP1 gene is widely used for molecular characterisation to classify FMDV serotypes into topotypes, lineages, and sublineages.

The global distribution of FMDV is characterised by endemic circulation of specific serotypes and genetic lineages within seven endemic pools of FMDV circulation [1, 2]. South Asia, comprising Bangladesh, India, Nepal, Bhutan, and Sri Lanka, is designated as Pool 2. In this region, serotypes O, A, and Asia1 have been reported historically, with serotype O being the most prevalent. All type O viruses in this region belong to the Middle East-South Asia (ME-SA) topotype. In contrast, type A viruses are predominantly of the ASIA topotype, particularly the G-VII lineage (also known as genotype 18) [3, 4]. In Bangladesh, serotypes O and A account for approximately 31–82% and 7–47% of outbreaks, respectively, while serotype Asia1 has been reported sporadically. Serotype C has not been detected in Bangladesh since 1992, and globally since 2004 [5–7]. Recent molecular epidemiological studies have shown the continuous emergence and circulation of new FMDV lineages in Bangladesh [8–11]. In 2013, O/ME-SA/Ind-2001e became the predominant serotype O sublineage, and in 2021, the O/ME-SA/SA-2018 sublineage was detected for the first time, subsequently showing a rapid increase in prevalence [12, 13].

For serotype A, A/ASIA/G-VII has remained the sole lineage detected since 2012. Additionally, the G-IX lineage of serotype Asia1 was first reported in Bangladesh in 2018 [14, 15]. Notably, in 2023, the A/ASIA/Iran-05/FAR-11 sublineage was identified for the first time in Bangladesh. The Iran-05 lineage was initially reported in Iran in 2003 and later spread eastward to Afghanistan and Pakistan, and westward to Saudi Arabia, Turkey, Jordan, and North Africa [16]. It has been considered endemic to Pool 3 regions for decades, including Western Asia and parts of Southern Asia (i.e., Afghanistan, Iran, and Pakistan) [17]. Thus, its detection in Bangladesh represents the first confirmed incursion of the Iran-05 lineage into Pool 2, signifying a critical epidemiological shift and potential regional dissemination risk [18].

Given the co-circulation in Bangladesh of multiple serotypes and emerging genetic variants (including O/ME-SA/Ind-2001e, O/ME-SA/SA-2018, and A/ASIA/G-VII) and the frequent reports of vaccine mismatch, continuous molecular surveillance and lineage-specific characterization are essential to inform effective control strategies and the selection of the most appropriate vaccines [19–22].

In this study, we aimed to investigate the molecular characteristics of 57 FMDV field isolates collected in Bangladesh between 2021 and 2023, focusing on serotype distribution, genetic lineages, and phylogenetic relationships. We report the first detection of the A/ASIA/Iran-05 lineage in Pool 2, providing critical insights for future vaccine selection, risk assessment, and regional disease control programs.

## Materials and methods

### Sample collection

FMD cases were reported to the Bangladesh veterinary authorities by field veterinarians. Eighty-nine clinical samples (tongue tissue) were collected from cattle showing clinical signs of FMD on farms in 16 districts between 2021 and 2023. Sample locations were mapped in QGIS 3.36.0 using the OpenStreetMap plugin, with Bangladeshi geographical admin layers downloaded from the Humanitarian Data Exchange. Clinical materials were placed in a viral transport medium (BD Diagnostics) and homogenized in Dulbecco’s modified Eagle’s medium (without fetal bovine serum) using a bead-based homogenizer. Subsequently, the samples were transported from the collection sites to the laboratory of the Central Disease Investigation Laboratory, where they were stored at −80 °C before being shipped to the FMD WOAH Reference Laboratory of the Animal and Plant Quarantine Agency (APQA) in the Republic of Korea.

### Virus isolation

The baby hamster kidney (BHK-21) cell line, obtained from the American Type Culture Collection (ATCC), was cultured for virus isolation in growth media that contained Dulbecco’s modified Eagle’s medium (Corning, NY, USA), 1 × antibiotic–antimycotic solution (Corning, NY, USA), and 10% fetal bovine serum (Corning, NY, USA). To isolate the virus, 20% of tissue homogenates were inoculated onto BHK-21 cell lines that were prepared a day before inoculation. Once 90% cytopathic effect (CPE) was observed, the cells and culture media were harvested and stored at −80 °C until analysis. When CPE was not observed, the cells and media were frozen and thawed, and the supernatant was centrifuged at 800 × *g* (Eppendorf, Hamburg, Germany) and inoculated onto freshly prepared cells for viral isolation.

### RNA extraction and 3D real-time reverse transcription (RT)-polymerase chain reaction (PCR)

Viral RNA was extracted from clinical samples (*n* = 89) and cell-isolated viruses (*n* = 57) using the Nextractor^®^ NX-48s viral NA kit (Genolution, South Korea), following the manufacturer’s instructions. Viral RNA was eluted in 40 µL of elution buffer and stored at −80 °C. According to the manufacturer’s instructions, total RNA was extracted from the collected samples (*n* = 89) using MagnaPure96 (Roche, Basel, Switzerland). Following extraction, 3D real-time RT-PCR was conducted, as previously described [23], to confirm the presence of FMD viral RNA.

### RT-PCR and sequencing

One-step RT-PCR was performed, and the VP1 coding region (1D) was amplified along with the flanking regions of the 1C and 2A regions according to published protocols [24] using FMD field isolates. Each amplicon (820 bp) was sequenced using a Big Dye Terminator v3.1 Cycle Sequencing Kit (Thermo Fisher Scientific, Waltham, MA, USA) with the same primers used for RT-PCR on an ABI 3730 DNA analyzer (Applied Biosystems, Foster City, CA, USA) following the manufacturer’s protocol.

### Sequence data collection and phylogenetic analysis

The VP1 coding sequences for 57 field viral isolates were compared with FMDV strains (serotype O [*n* = 45] and serotype A [*n* = 32]) and reference field strains (serotype O [*n* = 21] and A [*n* = 25]) collected from GenBank and the World Reference Laboratory for FMD (WRLFMD), Pirbright, UK, sequence database. Maximum likelihood trees were constructed using MEGA X using a general time-reversible substitution model [25]. The robustness of the tree topology was assessed using 1000 bootstrap replicates.

Additionally, the VP1 coding sequences of two A/ASIA/Iran-05 FMD isolates from this study, along with 872 VP1 sequences retrieved from the WRLFMD database, were used to construct time-scaled phylogenetic trees. A BEAST 1.10.4 [26] analysis was run using an uncorrelated relaxed clock model based on log-normal distributed branch rates, along with the GTR + Γ4 model of nucleotide substitution and the Bayesian skygrid coalescent tree prior [27]. The posterior estimates were obtained by running a Markov chain Monte Carlo (MCMC) for 200 million iterations, sampling every 20 000 states. The mixing and convergence of the MCMC chain were evaluated using Tracer 1.7.3 to ensure that the ESS of all parameters was > 200. The Maximum Clade Credibility tree was generated in Tree Annotator after discarding the first 10% of the chain as burn-in and then visualized in Figtree 1.4.4.

### Comparative analysis of VP1 coding proteins for A/ASIA/Iran-05 field viruses

Variability in the VP1 coding sequences of the two A/ASIA/Iran-05 FMDV field isolates (A/BAN5/2023 and A/BAN6/2023) was compared with those previously reported from Pakistan (*n* = 12) and Iran (*n* = 12) and visualized using CLC genomics Workbench 22.0.2 (Qiagen).

## Results

### FMD diagnosis and virus isolation

**Figure 1:**
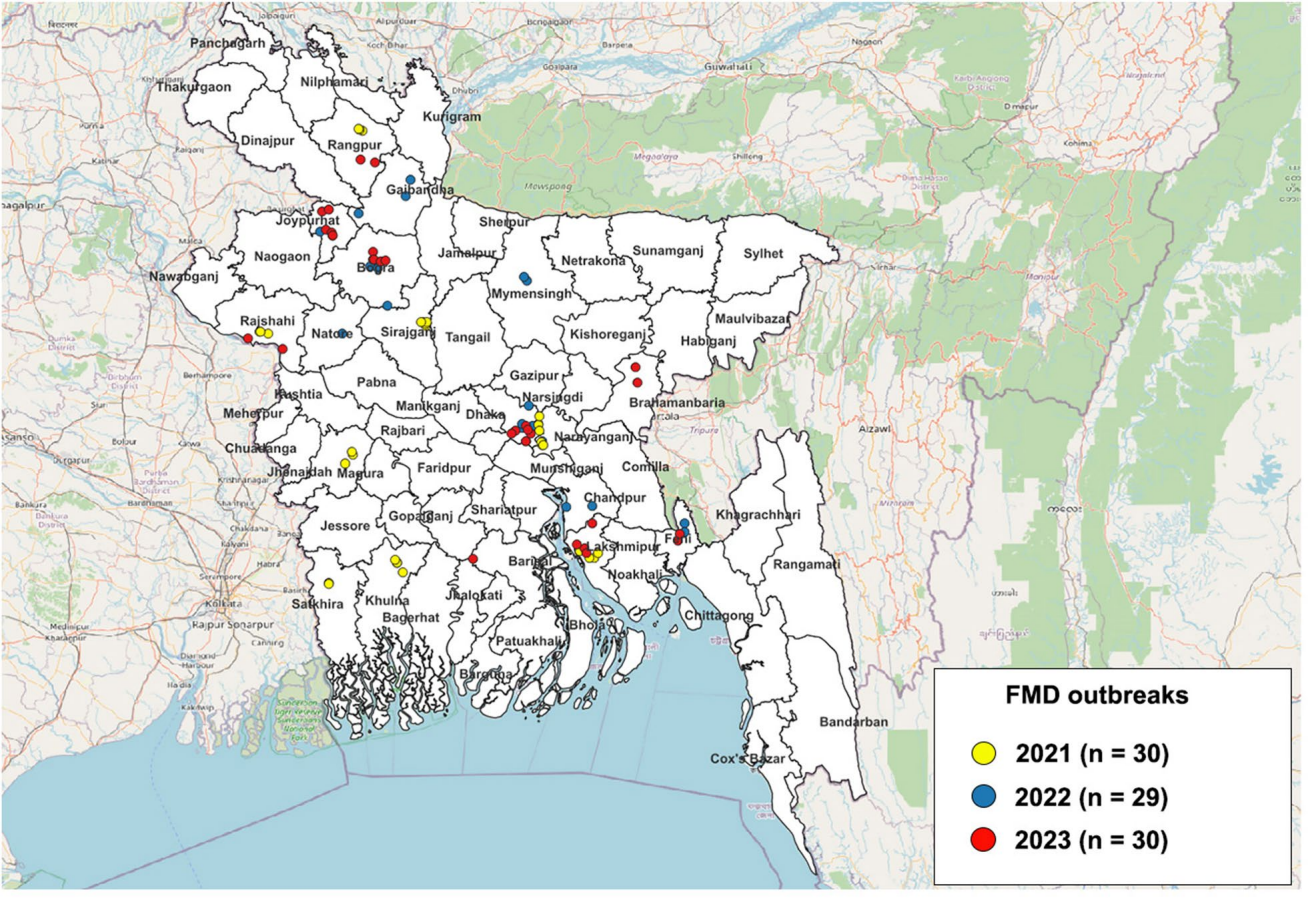
**Geographic locations of FMD cases detected in Bangladesh from 2021 to 2023**. The locations are marked with circles, with their colours defining the year of collection: yellow: 2021; blue: 2022; red: 2023.

Eighty-nine FMD cases reported in cattle were confirmed using 3D real-time RT-PCR in 16 districts of Bangladesh: Dhaka (*n* = 16), Lakshmipur (*n* = 13), Bogra (*n* = 9), Joypurhat (*n* = 8), Rajshahi (*n* = 7), Feni (*n* = 6), Sirajgonj (*n* = 6), Khulna (*n* = 4), Narayanganj (*n* = 4), Jhenaidah (*n* = 3), Rangpur (*n* = 3), Satkhira (*n* = 3), Brahmanbaria (*n* = 2), Gibandha (*n* = 2), Mymensingh (*n* = 2), and Chandpur (*n* = 1) (Figure 1). Fifty-seven out of the 89 clinical samples collected tested positive for FMDV by real-time RT-PCR (resulting in a Ct range of 17.3–34.2), with FMDV-specific CPE observed on BHK-21 cell culture (Table 1).

**Table 1:** **FMD field viruses isolated from FMD cases in Bangladesh (2021–2023)**

No.	Location	Date collected	Strain name	Lineages
1	Satkhira	04/03/2021	O/BAN1/2021	O/ME-SA/Ind-2001e
2	Satkhira	04/03/2021	O/BAN2/2021	O/ME-SA/Ind-2001e
3	Satkhira	04/03/2021	O/BAN3/2021	O/ME-SA/Ind-2001e
4	Dhaka	13/03/2021	O/BAN13/2021	O/ME-SA/Ind-2001e
5	Jhenidah	17/03/2021	O/BAN4/2021	O/ME-SA/Ind-2001e
6	Jhenidah	17/03/2021	O/BAN5/2021	O/ME-SA/Ind-2001e
7	Jhenidah	18/03/2021	O/BAN6/2021	O/ME-SA/Ind-2001e
8	Khulna	05/04/2021	O/BAN7/2021	O/ME-SA/Ind-2001e
9	Khulna	05/04/2021	O/BAN8/2021	O/ME-SA/Ind-2001e
10	Khulna	06/04/2021	O/BAN9/2021	O/ME-SA/Ind-2001e
11	Khulna	06/04/2021	O/BAN10/2021	O/ME-SA/Ind-2001e
12	Narayangonj	16/06/2021	O/BAN14/2021	O/ME-SA/Ind-2001e
13	Rangpur	18/07/2021	O/BAN21/2021	O/ME-SA/Ind-2001e
14	Rangpur	18/07/2021	O/BAN22/2021	O/ME-SA/Ind-2001e
15	Rangpur	19/07/2021	O/BAN23/2021	O/ME-SA/Ind-2001e
16	Rajshahi	12/08/2021	O/BAN24/2021	O/ME-SA/Ind-2001e
17	Rajshahi	12/08/2021	O/BAN25/2021	O/ME-SA/Ind-2001e
18	Rajshahi	12/08/2021	O/BAN26/2021	O/ME-SA/Ind-2001e
19	Sirajgonj	04/09/2021	O/BAN27/2021	O/ME-SA/Ind-2001e
20	Sirajgonj	04/09/2021	O/BAN28/2021	O/ME-SA/Ind-2001e
21	Sirajgonj	06/09/2021	O/BAN29/2021	O/ME-SA/Ind-2001e
22	Sirajgonj	06/09/2021	O/BAN30/2021	O/ME-SA/Ind-2001e
23	Lakshmipur	29/10/2021	O/BAN17/2021	O/ME-SA/Ind-2001e
24	Lakshmipur	29/10/2021	O/BAN18/2021	O/ME-SA/Ind-2001e
25	Lakshmipur	01/11/2021	O/BAN19/2021	O/ME-SA/Ind-2001e
26	Lakshmipur	01/11/2021	O/BAN20/2021	O/ME-SA/Ind-2001e
27	Dhaka	10/01/2022	O/BAN1/2022	O/ME-SA/SA-2018
28	Dhaka	16/01/2022	O/BAN2/2022	O/ME-SA/SA-2018
29	Dhaka	21/01/2022	O/BAN3/2022	O/ME-SA/SA-2018
30	Joypurhat	01/03/2022	O/BAN6/2022	O/ME-SA/SA-2018
31	Joypurhat	05/03/2022	O/BAN7/2022	O/ME-SA/SA-2018
32	Gibandha	10/03/2022	O/BAN8/2022	O/ME-SA/SA-2018
33	Gibandha	12/03/2022	O/BAN9/2022	O/ME-SA/SA-2018
34	Dhaka	08/05/2022	O/BAN12/2022	O/ME-SA/SA-2018
35	Chandpur	11/08/2022	O/BAN15/2022	O/ME-SA/SA-2018
36	Mymensingh	11/08/2022	O/BAN16/2022	O/ME-SA/SA-2018
37	Mymensingh	14/08/2022	O/BAN17/2022	O/ME-SA/SA-2018
38	Bogra	03/09/2022	O/BAN20/2022	O/ME-SA/Ind-2001e
39	Dhaka	10/10/2022	O/BAN21/2022	O/ME-SA/Ind-2001e
40	Dhaka	12/10/2022	O/BAN22/2022	O/ME-SA/Ind-2001e
41	Lakshmipur	31/10/2022	O/BAN26/2022	O/ME-SA/Ind-2001e
42	Dhaka	30/10/2022	O/BAN27/2022	O/ME-SA/Ind-2001e
43	Feni	10/11/2022	O/BAN28/2022	O/ME-SA/Ind-2001e
44	Feni	13/11/2022	O/BAN29/2022	O/ME-SA/Ind-2001e
45	Dhaka	05/03/2023	O/BAN3/2023	O/ME-SA/Ind-2001e
46	Dhaka	08/03/2023	O/BAN4/2023	O/ME-SA/Ind-2001e
47	Brahmanbaria	05/04/2023	A/BAN5/2023	A/ASIA/Iran-05
48	Brahmanbaria	05/04/2023	A/BAN6/2023	A/ASIA/Iran-05
49	Rajshahi	20/04/2023	O/BAN8/2023	O/ME-SA/Ind-2001e
50	Lakshmipur	26/04/2023	O/BAN18/2023	O/ME-SA/Ind-2001e
51	Joypurhat	26/04/2023	O/BAN10/2023	O/ME-SA/SA-2018
52	Dhaka	02/05/2023	O/BAN11/2023	O/ME-SA/SA-2018
53	Dhaka	04/05/2023	O/BAN12/2023	O/ME-SA/SA-2018
54	Joypurhat	06/05/2023	O/BAN19/2023	O/ME-SA/Ind-2001e
55	Joypurhat	08/05/2023	O/BAN21/2023	O/ME-SA/Ind-2001e
56	Feni	14/05/2023	O/BAN25/2023	O/ME-SA/SA-2018
57	Feni	14/05/2023	O/BAN26/2023	O/ME-SA/Ind-2001e

*All hosts were cattle; the sample type is tongue tissue

### Temporal trends in FMDV serotype and lineage circulation in Bangladesh from 2021 to 2023

The 57 FMDV field isolates were further analyzed to determine serotype and lineage distribution in FMD outbreaks reporting regions of Bangladesh between 2021 and 2023. The overall serotyping results revealed a predominance of serotype O (96.5%), with a smaller proportion of serotype A (3.5%), consistent with previous reports from 2021 [11] FMDV typing results obtained from the 57 isolates characterized only the O/ME-SA/Ind-2001e lineage as circulating in 2021 (*n* = 26), while during 2022 (*n* = 18), two different type O lineages were detected: O/ME-SA/Ind-2001e (39%), O/ME-SA/SA-2018(61%). In 2023 (*n* = 13), the FMDV lineage distribution shifted as follows: O/ME-SA/Ind-2001e (58%), O/ME-SA/SA-2018 (25%), A/ASIA/Iran-05 (17%, first-time detection in Bangladesh) (Table 2). Phylogenetic analysis revealed that the O/ME-SA/SA-2018 isolates from 2022 to 2023 were closely related to those reported in Bangladesh in 2021, but genetically distinct from the Indian O/SA-2018 viruses identified in 2018. The frequency of SA-2018 detection increased markedly from no detection in 2021 to 47% typing frequency over the following 2 years, indicating its rapid establishment in the Bangladeshi livestock population.

**Table 2:** **Temporal shift in FMDV lineage typing frequency in Bangladesh during the 2021–23 period, including the first detection of A/ASIA/Iran-05**

Year	Total isolates	O/ME-SA/Ind-2001e (%)	O/ME-SA/SA-2018	A/ASIA/Iran-05
2021	26	100	–	–
2022	18	39	61%	–
2023	13	58	25%	17%
Total	57	71	47%	3.5%

Despite the emergence of O/SA-2018 and the first detection of A/Iran-05, the O/ME-SA/Ind-2001e lineage remained the dominant FMDV lineage, comprising 71% of all isolates over the 3 years. Furthermore, this lineage exhibited evidence of increasing genetic diversity, although detailed data are not shown here.

### Comparison of homologies in the NT and AA sequence of the VP1 coding region and phylogenetic tree analysis

**Figure 2:**
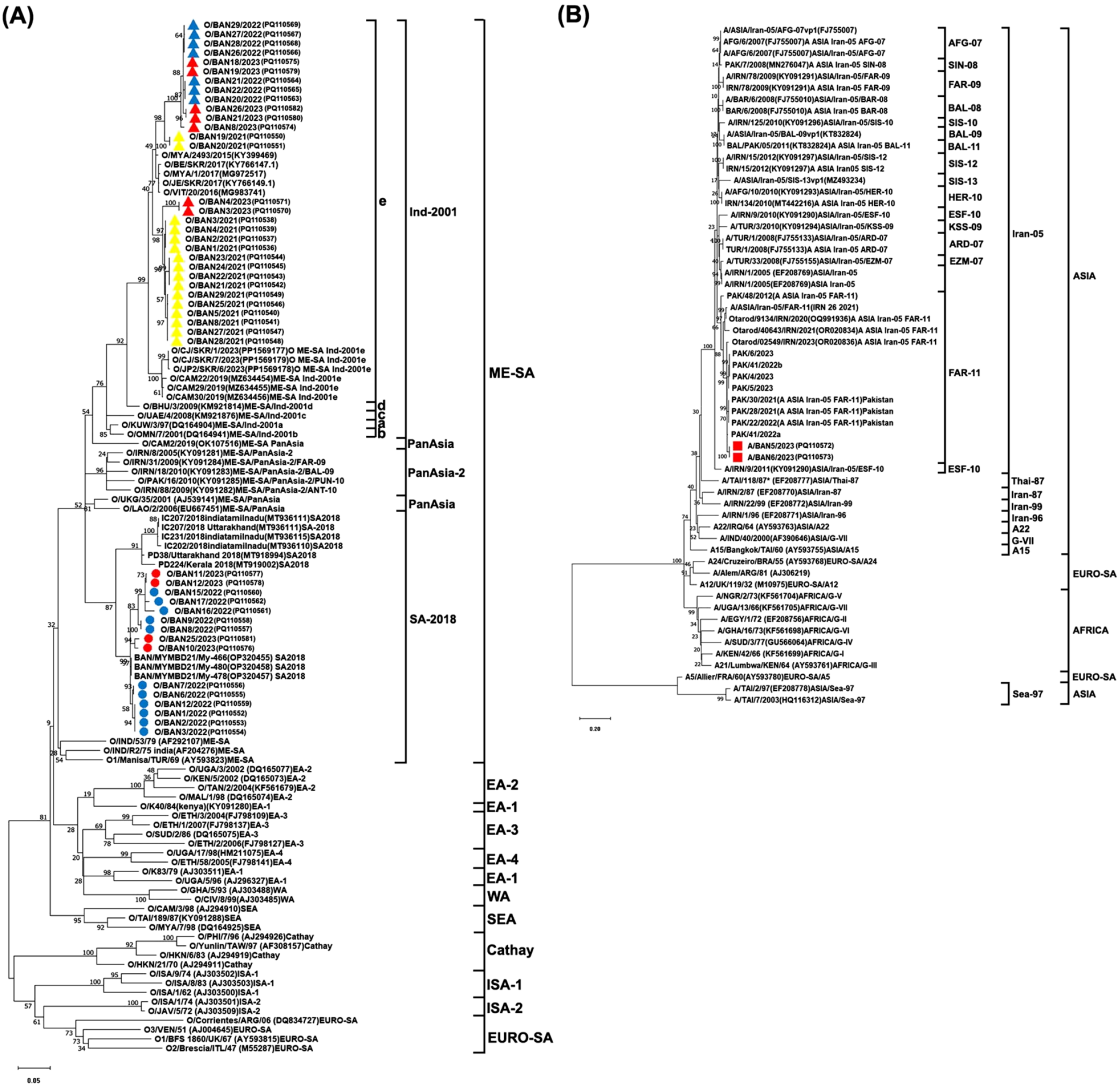
**Phylogenetic relationship of FMDV isolates detected in Bangladesh during 2021–23.** A maximum likelihood phylogenetic tree was reconstructed using the nucleotide sequence of the FMDV VP1 coding region to map the phylogenetic relationships between the (**A**) type O O/ME-SA/SA-2018 (marked with circles) and O/ME-SA/Ind-2001e (marked with triangles) FMDV lineages that caused outbreaks in Bangladesh and (**B**) A/ASIA/Iran-05 FMDV lineage (marked with squares) with reference isolates belonging to the same genotype circulating in neighboring regions. All Bangladesh field isolates are coloured to indicate the year of occurrence: 2021 in yellow, 2022 in blue, and 2023 in red.

Analyses conducted on all the 57 viral isolates sequenced showed no insertions or deletions within their VP1 coding regions. Phylogenetic trees were constructed using these sequences and including further FMDV sequences generated from global FMD genomic surveillance (Figure 2). The 57 field isolates were genotyped as follows: 40 were found to belong to the O/ME-SA/Ind-2001e FMDV lineage (26 from 2021, 7 from 2022, and 7 from 2023); 15 of the O/ME-SA/SA-2018 FMDV lineage (11 from 2022 and 4 from 2023); 2 of the A/ASIA/Iran-05 lineage (all collected in 2023) (Table 1). The between-sequence identity of the 40 Bangladesh FMDV isolates typed as O/ME-SA/Ind-2001e ranged from 92.2% (96.2) to 100% (100). By contrast, a sequence identity range of 91.7% (96.7) to 97.3% (100) was estimated for those FMDV isolates collected from other regions/countries (Table 3). O/ME-SA/Ind-2001e field isolates recovered from outbreaks in Bangladesh clustered in two different groups, showing relatively closer phylogenetic relatedness with FMDV isolates of Vietnam, Myanmar, and the Republic of Korea [between-sequences identity range of 93.9% (97.3) to 97.3% (100)].

**Table 3:** **Comparison of VP1 sequence identity estimated for the FMDV isolated from Bangladesh against reference strains**

Lineage	No. of isolates	Nucleotide (Amino acid) sequence Identity (%)	
		Bangladesh field isolates	Other countries’ field isolates^*^
O/ME-SA/Ind-2001e	40	92.2–100 (97.2–100)	91.7–97.3 (96.71–100)
O/ME-SA/SA-2018	15	94.7–100 (95.3–100)	89.8–99.0 (93.0–100)
A/ASIA/Iran-05	2	100 (100)	90.6–97.8 (93.0–98.6)

*For Bangladesh field isolates

For the 15 O/ME-SA/SA-2018 viruses isolated in Bangladesh, the between-sequence identity range in the VP1 coding region was estimated within 94.7% (95.3%) and 100% (100%). In comparison, a range between 89.8% (92.9) and 99.0% (100) was reported between Bangladesh isolates and those from other regions/countries. Field isolates collected between 2022 and 2023 were phylogenetically assigned to a clade grouping previous FMDV isolates collected in Bangladesh in 2021 [28–30]; sequence identity estimated at the within-clade level was in a range from 96.2% (96.7%) to 99.1% (100%). A clade grouping Indian field isolates from 2018 to 19 was distantly related, with a sequence identity range estimated at 89.8% (92.9%) to 94.4% (98.1%).

**Figure 3:**
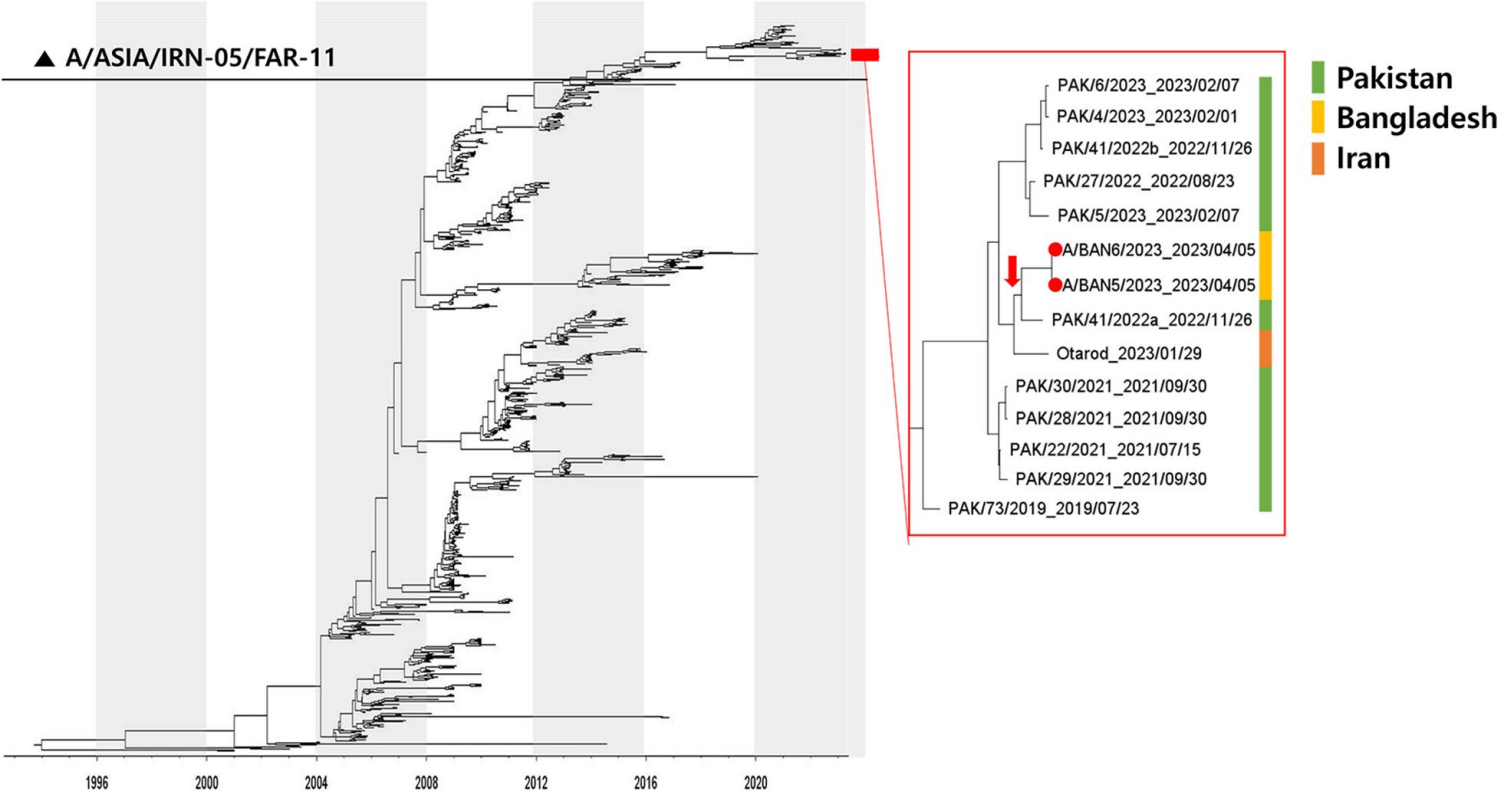
**Maximum Clade Credibility tree of the A/ASIA/Iran-05 FMDV lineages reconstructed using the FMDV VP1 coding sequences generated from isolates collected in Bangladesh during 2023 (red circle).** The tree maps their relationship with other A/ASIA/Iran-05 lineages prevalent in Pool 3. Slate blue bars represent the 95% High Posterior Density intervals for the timing of node ancestry. The box shows isolates from Pakistan in 2022, where MRCA (red-filled arrows) is shared with the FMD viruses isolated in Bangladesh during 2023 (red circle). The MRCAs of the A/ASIA/Iran-05 lineage from Bangladesh in 2023 (yellow bar), from Pakistan (green bar) in 2022–2023, and from Iran in 2023 (orange bar) are further highlighted.

Two FMD isolates belonging to serotype A and collected in 2023 (A/BAN5/2023 and A/BAN6/2023) were found to be 100% identical in their VP1 coding region. These sequences were genotyped as A/ASIA/Iran-05, FAR-11 sublineage, and phylogenetically placed within a clade that also comprises A/ASIA/Iran-05^FAR−11^ viruses circulating in Pakistan during 2021–2023 [sequence identity within 96.7% (97.6) and 97.8% (98.6)]. The timing of the most recent common ancestor (MRCA) of these Bangladesh isolates was estimated at March 2022 (95% BCI September 2021 to November 2022), with this MRCA shared with a virus detected in Pakistan during 2022 (PAK/41/2022a) (Figure 3). An Iranian virus detected in 2023 was found to be basal to this phylogenetic clade (Otarod/02549/IRN/2023)

**Figure 4:**
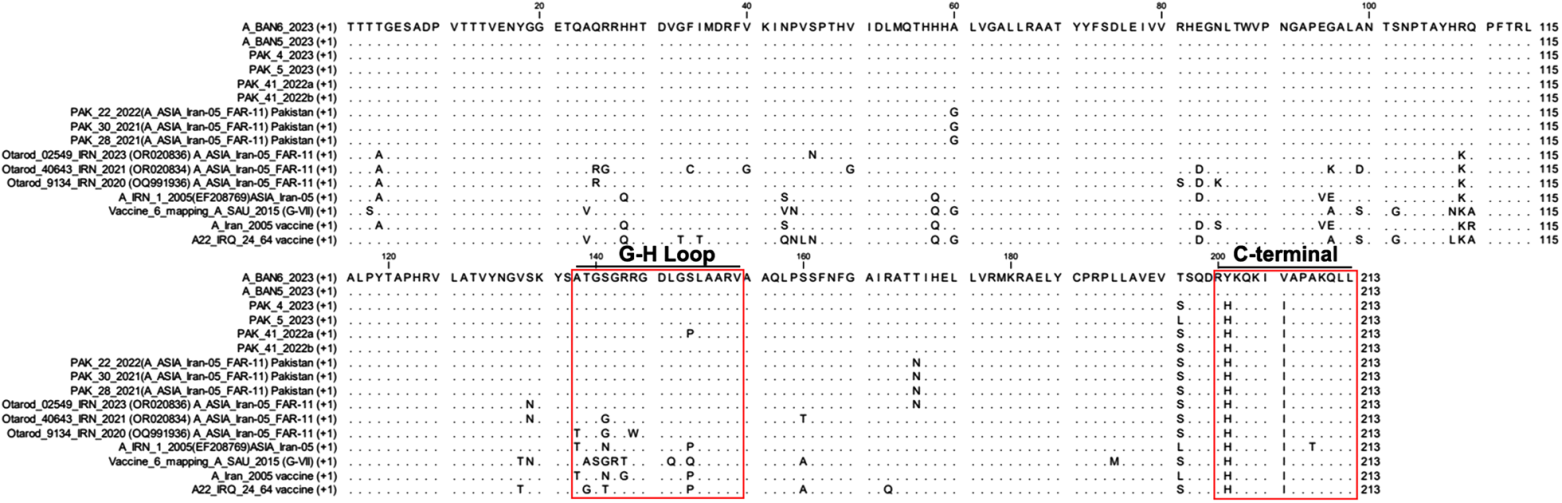
**Alignment of VP1 amino acid sequences.** Alignment was performed for the Bangladesh FMDV field isolates (A/BAN5/2023 and A/BAN6/2023) belonging to the A/ASIA/Iran-05 lineage, reference isolates, and vaccine strains (A/G-VII/2015, A/IRN/05, and A22/IRQ/24/64). The GH loop (residues 138–154) and the C-terminus region of the VP1 coding region (residues 200–213) belonging to the antigenic site are shown. Dots indicate conserved AAs, and letters indicate AA substitutions. The red box defines antigenic sites.

### Variability in the VP1 coding region of the A/ASIA/Iran-05 FMDV lineage

From the comparative analysis of the deduced AA sequence of the VP1 coding region, three substitutions specific to the Bangladesh field isolates were identified: Y201H and V206I, in the C-terminus region; T196S or T196L, in the region close to 5’end of the C-terminus region, which differentiated them from those viruses detected in Pakistan and Iran during 2022 and 2023 (PAK/41/2022a, PAK/41/2022b, PAK/4/2023, PAK/5/2023 and Otarod/02549/IRN/2023) (Figure 4). Three random AA substitutions were also found within the G-H loop. Comparison of the AA sequences of the VP1 coding region between FMDV vaccine strains (A/G-VII/2015 and A/IRN/05) and the Bangladesh field isolates revealed specific substitutions (T139A, G140S, S141G, G142R, R143T, L147Q, and S149Q) within the G-H loop for the vaccine strain A/G-VII/2015 and specific substitutions (A138T, S141T, R143G, and S149Q) within the G-H loop for A/IRN/05. We also found that only the prototype strain (A/IRN/1/2005) was substituted in the C-terminal region with A209T. Conversely, the other vaccine strains and outbreak field isolates all had the substitution at the same position.

## Discussion

In this study, we provide updated molecular insights into the circulation of FMDV in Bangladesh between 2021 and 2023, highlighting dynamic changes in serotype distribution and genetic lineage diversity. Serotype O remained predominant throughout the study period, accounting for over 70% of all field isolates collected [11]. Within serotype O, the O/ME-SA/Ind-2001e lineage continued to circulate widely; however, the emergence and subsequent expansion of the O/ME-SA/SA-2018 sublineage since 2021 signify a shift in the local viral population structure. The increasing detection frequency of O/SA-2018, particularly its dominance over O/Ind-2001e in 2022, may reflect changes in FMD epidemiological dynamics. These changes could be driven by altered transmission pathways between regions, likely influenced by cross-border livestock movements and variations in livestock trade pricing [30–34]. Collectively, these findings underscore the importance of regular lineage-specific monitoring, even within a single serotype.

The study period was limited to 3 years; however, analysis of the FMDV collection revealed significant temporal shifts in lineage prevalence [11, 30]. These findings provide valuable insights that may serve as a proxy for understanding the evolving epidemiological dynamics of FMD in neighboring regions, potentially reflecting changes in risk pathways [10, 35]. However, long-term surveillance with broader spatial coverage is needed to better characterize the complex epidemiology of FMDV in Bangladesh, map potential pathways for novel virus introduction from neighboring regions, and identify within-country hotspots of virus persistence [36]. To address this, we have initiated ongoing molecular surveillance using newly collected field samples from 2024 onwards, which will support continued monitoring of FMDV evolution in Bangladesh and enable early identification of emerging lineages.

A notable finding of this study is the first detection of the A/ASIA/Iran-05 lineage in Bangladesh, marking its initial incursion into the FMD endemic Pool 2. Historically, the Iran-05 lineage has been predominantly found in Pool 3, particularly in Iran, Pakistan, and the broader Western Asia region [35]. The detection of two genetically identical isolates (A/BAN5/2023 and A/BAN6/2023) in Brahmanbaria, eastern Bangladesh, close to the Indian border, suggests a likely transboundary introduction. Notably, A/ASIA/Iran-05 has not yet been reported in neighboring countries in Pool 2, including India, Nepal, Bhutan, and Sri Lanka. Phylogenetic analysis revealed a close relationship with PAK/41/2022a, suggesting a potential epidemiological link to Pakistan. However, given the underreporting of FMD cases and the opportunistic nature of case sampling in FMD endemic regions, the precise route of introduction remains speculative. These findings highlight the importance of collaborative regional surveillance and transparent sharing of genomic data to accurately track viral movements across borders.

While the primary focus of this study was the genetic characterization of circulating FMDV strains, AA substitutions identified in the VP1 protein, particularly H202Y and V206I within the C-terminal region of the A/BAN5/2023 and A/BAN6/2023 isolates, may have antigenic implications. These residues are outside the RGD motif; the C-terminal region is known to contribute to conformational neutralizing epitopes. Notably, these substitutions were unique to the Bangladeshi isolates and absent in both vaccine strains and other regional field viruses.

Currently, Bangladesh uses domestically produced vaccines and imported serogroup O, A, and Asia1 vaccines, among others [10, 11, 30, 37]. However, newly discovered field isolates, particularly those first identified within Bangladesh, such as A/ASIA/Iran-05, may not be antigenically fully matched to the vaccine strains in use.

Without updating vaccine strain selection and ensuring regular antigenic match, the effectiveness of existing vaccines against these evolving lineages may diminish. Therefore, proactive assessment of vaccine-field strain compatibility is crucial, and efforts to integrate regional field isolates into the national vaccine development pipeline should be prioritized. In this study, a two-dimensional virus neutralization test (2D-VNT) was not performed due to difficulties in vaccine and vaccine strain introduction in Bangladesh. However, further studies are planned to determine whether the vaccine currently used in Bangladesh provides protective efficacy against the novel variant of the A/ASIA/Iran-05/FAR-11 lineage.

In conclusion, this study demonstrates the expanding genetic diversity of FMDV in Bangladesh, including increased prevalence of the O/ME-SA/SA-2018 lineage and the new detection of the A/ASIA/Iran-05 lineage. These findings underscore the ongoing evolution and transboundary movement of FMDV within the South Asian region, providing critical information for establishing national and regional disease control programs. We emphasize the need for continuous molecular surveillance, antigenic profiling, and vaccine matching to respond to emerging variants proactively. To this end, we will continuously monitor recently isolated FMD field isolates in Bangladesh to support genomic surveillance and contribute to the development of evidence-based control strategies.
